# Family or otherwise: Exploring the impact of family motivation on job outcomes in collectivistic society

**DOI:** 10.3389/fpsyg.2023.889913

**Published:** 2023-03-02

**Authors:** Samina Yaqoob, Muhammad Ishtiaq Ishaq, Mamoona Mushtaq, Ali Raza

**Affiliations:** ^1^Dr Hasan Murad School of Management, University of Management and Technology, Lahore, Pakistan; ^2^School of Management Sciences, Quaid-i-Azam University, Islamabad, Pakistan

**Keywords:** family motivation, collectivism, psychological meaningfulness, job performance, intent to leave

## Abstract

The motive of the current research is to determine the influence of family motivation on intent to leave and job performance using self-determination theory. Moreover, this study also explores the moderating role of collectivistic culture and the mediating role of psychological meaningfulness on the relationship between family motivation and work outcomes. The data (*N* = 175) were collected from paramedical staff working in Pakistani public hospitals, and data was analyzed using PROCESS method. The findings revealed that family motivation enhanced employee job performance and lessened employees’ intent to leave. At the same time, family motivation and psychological meaningfulness are stronger in highly collectivistic cultures compared to less collectivist cultures. This study extends the investigation of the newly developed construct of family motivation by focusing on psychological meaningfulness and collectivistic culture. Moreover, this study is the first to introduce psychological meaningfulness as a mediator and collectivistic culture as a moderator for the relationship between family motivation and employee job outcomes. This study provides several critical insights for the hospitals by exploring the importance of family motivation as a potential motivational resource for maintaining high employee job-performance levels and lessening the intent of employees to leave.

## Introduction

Every human being performs in an exceptional context that influences their confidence and morale about achieving family-related and work-related goals. However, research has found significant variability in how human beings anticipate balancing or integrating these two aspects of life (Gerson, 2010). The family is one of the essential components of social relationships in all cultures, and it is challenging to comprehend a culture living in the absence of the family. Minuchin et al. (1981) have posited that the family is the natural milieu for both healing and growth. This natural setting evolves and strengthens the interactions among family members. Additionally, they argued that “a viable form of family structure is needed to perform the family’s essential tasks of supporting individuals while providing a sense of belonging” (p. 112). In this scenario, all family members perform in a symptomatic way, developing attitudes that help prioritize the family’s needs, keep the family away from harm, and protect the family’s future. Altogether, this suggests that an individual endorses work-related and family-related goals to achieve these goals as they develop, but does so differently in different developmental phases (Ahmad et al., 2023).

Family motivation, understood as the aspiration to work to support one’s family (Menges et al., 2017), is an integral aspect of the workplace that inspires employees to perform effectively and efficiently to support their families in different workplace settings (Tariq and Ding, 2018); it develops with greater intensity when the employee’s family is walking a financial tightrope. To date, numerous studies have portrayed its detrimental effects on multiple employee-related outcomes including performance, intent to leave, and work-life conflicts (Lee et al., 2018; Morgan et al., 2018). However, only limited research has attempted to unpack its positive impact on employee job outcomes (Menges et al., 2017; Tariq and Ding, 2018), although such outcomes are common phenomena, linked to every individual who belongs to a family-oriented society.

The studies of Schwartz et al. (2012) and Schwartz and Bilsky (1990) presented family as a universal prize value in most of the cultures. The previous researches strongly supported the influence of family on motivation to work (Tariq and Ding, 2018). When the job is exciting, the employees felt internally motivated to work hard to get higher job performance (Piccolo and Colquitt, 2006). This intrinsic motivation fosters employees to work longer, smarter, harder, and productive (Gagné and Deci, 2005). But this appreciation is not linked to many jobs that enable intrinsic motivation like agriculture, service, and manufacturing sectors. In such jobs, the employees have few or no preferences in schedules, work methods, decisions, and tasks (Morgeson and Humphrey, 2006; Davis, 2010), especially in developing countries like Pakistan. In this situation, the employees felt deprived of autonomy, which is among highly contributors to intrinsic motivation in both organizational researches using the job characteristics model (Hackman, 1980) and psychological researches using self-determination theory (Deci and Vansteenkiste, 2004).

Even though one of the values that pushes many employees to work is the importance of supporting their families, few studies have examined family motivation to support their families (Brief et al., 1997). Zhang et al. (2020) argue that family motivation is a double-edged sword that has both debilitating and energizing influence on work-related outcomes. Therefore, the current research aims to understand the role of family motivation and why employees associate themselves in organizations that cause long duty hours, workload, burnout and stress. Frankl’s (1984) also propose the role of family in working environment as “he who has why to live can almost bear anyhow.” Moreover, the majority of studies on family motivation have concentrated on negative organizational, interpersonal, and individual work outcomes (Amstad et al., 2011). Taking these caveats into consideration, this study proposes that family motivation affects individual work outcomes both positively and negatively, simultaneously among paramedical staff of public hospitals in Pakistan. This research also explores the moderating role of collectivistic culture and the mediating role of psychological meaningfulness on the relationship between family motivation and work outcomes.

### Literature review

#### Self-determination theory

Individuals perform various responsibilities and hunt different goals throughout their interaction with a multitude of important life domains. However, not all domains are heading in the same direction – some are depleting, while others may be fulfilling. What establishes whether individuals feel energetic or ecstatic in a given domain? The self-determination theory (SDT) exhibits the significance of the psychological need for satisfaction on positive work behaviors and attitudes (Deci and Ryan, 1980; Deci et al., 1999). Numerous theories conceptualize changing work motivation largely in terms of quality. In contrast, SDT proposes two different types of motivation that can direct employees’ volitional motivation: intrinsic motivation and extrinsic motivation (Deci and Ryan, 2012). Intrinsic motivation (autonomous actions) performs tasks because of interest or inherent inclination, while extrinsic motivation (controlled actions) performs activities to attain additional rewards or avoid punishments (Gagné and Deci, 2005). Intrinsic motivation is exhibited in an employee’s natural propensity to learn and to seek challenges and novelty for their own sake. On the SDT continuum, the highest end is autonomy (having the liberty to make decisions actively), whereas the lowest end is amotivation (lacking intention to act; Deci and Ryan, 2000; Deci and Vansteenkiste, 2004).

External regulation is the most controlled form of extrinsic motivation that is maintained and initiated by the likelihood of external forces on employees, such as punishment or rewards (Gagné and Deci, 2005), and linked with negative outcomes (Ryan and Deci, 2000). Introjected regulation is a somewhat less-controlled form that indicates that internal punishments and rewards drive employee behavior. In this type of motivation, an employee is interested in preventing self-conscious emotions, like self-criticism, guilt, and shame, and in attaining positive appraisals and self-related effects (Koestner and Losier, 2002). Identified regulation is a strong, autonomous form; employee behavior is compatible with their values and goals. In summary, SDT classifies five motivation types in ascending order – amotivation, external regulation, introjected regulation, identified regulation, and intrinsic motivation (Ryan and Deci, 2000).

SDT has been examined in various empirical studies. Findings indicate that autonomous motivation is usually linked with positive outcomes such as job satisfaction, commitment, persistence, self-regulation, and performance (Nie et al., 2015; Deci et al., 2017). The inconsistencies in behaviors and values across cultures have led researchers to consider well-being, motivation, and social integration issues through cultural lenses. Some researchers (e.g., Chirkov et al., 2003) argue that the behaviors with autonomous motives nurture greater well-being, performance, and persistence when compared with controlled motives. However, some researchers propose that employees from collectivistic cultures put less importance on autonomy and prefer interdependence; they may not attain the benefits that Western countries extract from autonomy support (Schwartz, 1994; Kitayama et al., 2004).

The academic literature debates the influence and prevalence of extrinsic and intrinsic motivation in the workplace (Sansone and Harackiewicz, 2000). Some researchers argue that there is less of a chance that intrinsic motivation occurs in the workplace when compared to other realms because of inherent concentration on recognition at work (Baard, 2002). On the other side, Ryan and Deci (2000) argued that additional rewards given to the assigned tasks as extrinsic motivation but could not overlook intrinsic motivation. As SDT implies, extrinsic motivation comprises working exclusively to avoid punishments or obtain rewards (Gagné and Deci, 2005); family motivation encompasses ascertaining work as a core value or incorporating work with an entire values system. Nevertheless, there is a lack of research in Pakistani scenarios, especially among the paramedical staff of Pakistani hospitals.

#### Family motivation and job outcomes

Menges et al. (2017) define family motivation as an aspiration to do a employment to support his/her family members. They argue that family motivation acts divergently in case of low intrinsic employee motivation that would keep them aligned towards positive job outcomes. When work has no meaningful impact in the workplace, then family motivation acts as a powerful source of motivation and meaningfulness in the workplace (Rosso et al., 2010; Menges et al., 2017; Lin et al., 2020). Ryff and Singer (1998) propose that family is one of the prime facets among the fundamental sources of the meaning of life because individuals are motivated to serve their families (Menges et al., 2017). Hence, individuals are more inclined towards their work. Additionally, it can metamorphose uninteresting work into meaningful work (Tariq and Ding, 2018) since it aligns the behavior of employees with another prime motive of serving family members.

Family motivation is an under-researched concept and one of the potential sources of inspiration and motivation for working people in a different context (Menges et al., 2017; Tariq and Ding, 2018; Erum et al., 2020). There are individuals whose jobs are toxic, low paying, boring, without an opportunity for advancement, and without monetary benefits or bonuses, but still, they are types of jobs (Nawaz et al., 2022). Motivation is either extrinsic or intrinsic (Gagné and Deci, 2005), but if both motivational forces are absent, why do people still perform their jobs? The answer to this question is the inspiration for serving their families (Menges et al., 2017). Family motivation may act as a third motivational force for an employee, as Clark (2017) argues that family motivation can exhibit both motivational forms (intrinsic or extrinsic motivation), depending on values. For instance, if a family is a top priority, then it serves as an intrinsic force, but family obligation and pressure may turn it into an extrinsic force. Employees find the single powerful motivational source behind themselves, that is, “family” (Menges et al., 2017; Lin et al., 2020; Zhang et al., 2020), in the absence of intrinsic and extrinsic motivational factors.

SDT (Ryan and Deci, 2000) suggests that the three basic psychological needs that are essential for the wellness of human beings are relatedness, autonomy, and competence. This theory emphasizes close relationships and the well-being of these relations and is more inclined towards a family-oriented perspective (David, 2016). In his study Crosby (1984) argues that if family life is positive, then it acts as a shock absorber and chunk the effect of disappointment at work. The family and work domains are interdependent and complementary (Gutek et al., 1981). The valued purpose of an employee’s life serves as an alternative source of motivation in the absence of intrinsic motivation (Vroom, 1964). In this context, the prime motivation of employees is serving their families. Family motivation strongly predicts meaningfulness found in performing job duties successfully, because the pay they get from their jobs will be used to fulfill their family responsibilities. This picture is more salient in a collectivistic culture like Pakistan (Islam, 2004).

In developing countries like Pakistan, employees work primarily to support their families because any action is taken on behalf of family well-being (Islam, 2004). Family motivation provides an employee with a sense of identification, and in the case of low intrinsic motivation, his/her efforts to doing a job are greater (Rosso et al., 2010). An employee with low intrinsic motivation does not concentrate on a job (Zhang and Bartol, 2010), which leads to counterproductive behavior. Still, the scenario of serving a dependent at home charismatically alters the psychological experience of a job (Tariq and Ding, 2018). Additionally, they claimed that family love is a prime source and plays a fundamental role in an individual’s life, with a significant impact on job efficiency and effectiveness.

On the other side, in the presence of intrinsic motivation, employees naturally drag towards work (Grant, 2008), ready to put all their efforts into a job because they find the work enjoyable and interesting. However, it is not the same for every employee who is intrinsically motivated and happily bears all obstacles during his work (Frese and Fay, 2001) because intrinsic motivation is crippled for low-level jobs (Deal et al., 2013). In this essence, family motivation (Menges et al., 2017) can be suggested to mitigate an individual’s intent to leave (Vigoda, 2000) because they are extrinsically motivated to perform their work to their optimal capability, which in turn, boosts employee growth in terms of job performance (Williams and Anderson, 1991; Erum et al., 2020).

Previous literature proposes that employees give more care and consideration to their families because of biological associations, kinship, and bonding (Korchmaros and Kenny, 2001). Hence, it is proposed that employees with strong family motivation are expected to unveil spirit at work and stay positive and motivated, which will eventually decrease their intent to leave a job. Moreover, when a job mainly relates to realizing family obligations, it may also create a low intent to leave – a possible connection that is overlooked in developing countries like Pakistan, where unemployment is increasing. Based on SDT, family motivation is based on a controlled mechanism where an employee is working because of societal pressure or financial pressure to give benefits to his/her family members. This study proposes that family motivation is a potential motivator for an employee’s job performance (Menges et al., 2017; Tariq and Ding, 2018) and an inhibitor of their intention to leave a job (Menges et al., 2017; Lee et al., 2018; Tariq and Ding, 2018). Therefore, H1 proposes as

*H1: *Family motivation positively associates with job performance and negatively influences intention to leave.

#### Family motivation and psychological meaningfulness

There has been extensive research on psychological meaningfulness at work, and it has been recognized as a vital psychological state for an employee’s productivity, experience, motivation (Hackman, 1980; Liu and Zhou, 2018). Kahn (1990) refers to psychological meaningfulness as “a feeling that one is receiving a return on investments of one’s self in a currency of physical, cognitive, or emotional energy. People meaningfulness when they worthwhile, useful, and valuable—as though they [make] a difference and not taken for granted” (p.704).” Researchers, before and after Kahn’s (1990) study, highlight the proficiency of psychological meaningfulness as an influential and important condition that influences employees’ work behaviors (Liu and Zhou, 2018; Martela and Riekki, 2018).

Silbert (2012) suggests that meaningfulness can extend anywhere in one’s life, from providing necessities to family to beyond higher-order meaning. Martela and Riekki (2018) finds that meaningfulness is an important variable for millennials that drives them to perform harder and remain loyal to their organizations. Clark (2017) states that the alignment of a job with an individual’s values significantly influences his/her motivation. v posits that if there is an absence of intrinsic motivation in employees, then an esteemed purpose serves as an alternative source of motivation. Researchers argue that meaningfulness is a powerful source of energy (Pink, 2009), and family motivation is itself a vital source of meaningfulness in one’s life (Ryff and Singer, 1998) that makes work more valuable for employees. Baklaieva (2016) suggests that if employees find their assigned job psychologically meaningful, they produce positive outcomes, as reported by Menges et al. (2017) and Tariq and Ding (2018). Therefore, psychological meaningfulness motivates employees to engage in work that financially supports their families. Hence, H2 proposes as:

*H2: *Family motivation positively relates to the employee’s psychological meaningfulness.

#### Psychological meaningfulness and job outcomes

Pratt and Ashforth (2003) suggest that the extent to which individuals associate meaningfulness to their work influences their motivational level, boosting and supplementing a sense of growth with productive outcomes. Meaningfulness is considered to be related to performing actions that are voluntarily and consistent with individual values (Weinstein et al., 2012), and individuals effectively perform these actions (Baumeister, 2018). However, psychological meaningfulness is a subjective concept and presents with different behaviors in employees. Researchers (Ashmos and Duchon, 2000) elaborate that when individuals find meaning in their jobs, they are better able to align with a firm’s values. Additionally, adverse effects can be eliminated or prevented by finding meaning (Schadenhofer et al., 2018) in many over-stressed professions, such as paramedical staff. Smith and Aaker (2013) find that employees who exhibit meaningfulness at work are entitled as “givers.” Researchers (Van Wingerden and Van der Stoep, 2018) investigate the positive association between work meaningfulness and employee job performance. Jung and Yoon (2016) note that employees who find psychological help (family members) and more work meaning are likely to perform better. Janik (2015) claim that low psychological meaningfulness in one’s job has significant positive effects on an individual’s intent to leave. Another study (Li et al., 2011) finds that maintaining a perception of meaningfulness in one’s work ultimately reduces the intent to leave a nursing job. By adopting a meaningful mindset (Smith and Aaker, 2013), that is, a self-determining behavior, individuals make their own decisions, seek relatedness, and orient themselves towards a great purpose by finding meaning in their jobs. They also contribute towards family (Menges et al., 2017), show better performance (Jung and Yoon, 2016), and rarely intend to leave their jobs (Li et al., 2011; Janik, 2015). Thus, with this comprehension, H3 proposes as:

*H3: *Psychological meaningfulness positively relates to employee job performance and negatively relates to employees’ intention to leave.

#### Psychological meaningfulness as a mediator In family motivation-job outcomes relationships

Previous studies confirmed the mediating role of psychological meaningfulness for multiple work-related antecedents and consequences (Woods and Sofat, 2013; Cai et al., 2018; Chaudhary and Panda, 2018). As mentioned in Kahn’s (1990) explanation of meaningfulness, when employees feel eloquent, advantageous, and treasured, they perceive that they are making a change and are not being taken for granted. Meaningfulness (Kahn, 1990) denotes the perception that individuals want to take pride in what they perform for a living. Family motivation (Menges et al., 2017), as a backbone, helps them relate their work to their family, which ultimately provides positive outcomes.

Using SDT, the mediating role of psychological meaningfulness in the relationships of family motivation and job outcomes was endorsed. SDT explains that every individual has the right to determine one’s directions and make any decision in life (Gagné and Deci, 2005). When employees are autonomous and extrinsically motivated, they identify values as discretionary objectives. Within this umbrella, employees feel more freedom (identification regulation) because their behaviors are more aligned with their motives and identities. Gagné and Deci (2005) elegantly explains this with the example of nurses, who carry their profession regardless of unpleasant tasks, understanding the importance of their share in the well-being of patient health and the spirit of autonomy because they give value, comfort, and satisfaction to their patients. A similar research study elucidates that family members are responsible for providing support to their offspring to fulfill their psychological needs (Grolnick and Ryan, 1989). Nudging the grounds of SDT, Hedges (2017) argues that paramedical staff, regardless of their difficult job schedule/routine, carry their occupation with an inner sense of purpose. The staff feels the importance of their contributions to the well-being of their family members with the strong essence of their relationships (Deci and Vansteenkiste, 2004) because of family responsibilities (Menges et al., 2017).

This study rationalizes that when an individual is not putting effort into completing tasks, then family motivation acts as a source of motivation that gives a sense of meaningfulness in the assigned tasks (Arnoux-Nicolas et al., 2016), which ultimately influences positive job outcomes. As mentioned by Deci and Vansteenkiste (2004), people-orientation develops towards subsequent work activities with the motivation to contribute their support for loved ones or family members. Researchers (Jung and Yoon, 2016) report that employees who can find more meaning in their work (i.e., fulfill their family’s financial needs) and have psychological support from their loved ones perform better than others. Van Wingerden and Van der Stoep (2018) find a positive relationship between employee meaningfulness and job performance. Li et al. (2011) suggest that maintaining a sense of meaningfulness reduces an employee’s intent to leave. Low psychological meaningfulness in one’s job has a positive, direct link with intent to leave (Janik, 2015). Employees with a high orientation of family motivation are probably more engaged in high performance at work, with less inclination to leave their jobs. The root cause of these favorable job outcomes is the meaningfulness of the work, which is an intervening mechanism through which family motivation acts more vigorously and increases the meaningfulness of work. Subsequently, the meaningfulness of the work leads towards high performance and less intent to leave. We rationalize from Deci and Vansteenkiste’s (2004) standpoint rationalizes that employees in Pakistan capture their inner sense of meaning in their work from the powerful source of family motivation, which triggers employees’ job performance (Menges et al., 2017; Van Wingerden and Van der Stoep, 2018) and dampens their intent to leave (Tariq and Ding, 2018). Therefore H4 and H5 propose as:

*H4: *Psychological meaningfulness mediates the relationships between family motivation and job performance.

*H5: *Psychological meaningfulness mediates the relationships of family motivation and employee intent to leave.

#### Moderating role of collectivism

A collectivistic culture is a psychological orientation that mirrors the extent to which employees care about others or the group in which they belong (Dorfman and Howell, 1988). To determine the culture of a nation, researchers usually use Hofstede’s cultural model that consists of five dimensions. The individualism–collectivism dimension is highly significant and accounted for 52% in examining cultural differences (Engelen and Brettel, 2011; Ishaq et al., 2022) and considered the most trusted in many disciplines. Pakistan’s individualism–collectivism scale score is 14, which makes Pakistani culture highly collectivistic based on long-term commitment, closed and extended family relationships, and society. This behavior usually supersedes some societal norms, regulations, and rules to support one’s family or group. The family and kinship structure manifested the collectivistic culture of Pakistan; these structures have a central position and are cohesively integrated within the system. Mutual obligation and loyalty – any action on behalf of the pursuit of family well-being – are prioritized over laws and rational or professional codes of conduct (Islam, 2004). Similarly, parents and elders occasionally sacrifice their personal needs for the favor of their children or to support the necessities of loved ones, eventually depicting self-determined behavior (Martela and Riekki, 2018).

According to Baumeister (2018), meaning is culturally constructed. What makes a life meaningful is not simply a process of interaction but also participating in a cultural system that contains norms, values, and shared information. Social relationships and meanings are positively linked with each other (Baumeister, 2018; Ahmad et al., 2022). Islam (2004) proposes that in a collectivistic culture, people give priority to their families and perform activities on their behalf. Positive self-meaning can act as a resource for employees to help them realize their full potential in the workplace (Dutton et al., 2010).

In a collectivistic culture, people tend to honor family values and behave accordingly (Islam, 2004; Tan, 2012). Hence, in a collectivistic culture, family motivation is a more salient feature that generates work meaningfulness because individuals feel more responsible for contributing to their family. In this regard, collectivistic culture is proposed to be considered a potential moderator for this study for the relationship between family motivation and psychological meaningfulness. Moreover, family motivation acts more saliently in the presence of cultural collectivism, which encourages an individual’s job performance and discourages their intent to leave (Menges et al., 2017; Tariq and Ding, 2018). So, H6 proposes as:

*H6: *The collectivist culture will moderate the relationship between family motivation and psychological meaningfulness such that the relationship is stronger at a higher level of collectivism.

## Method

### Sample and procedures

The sample of the study was paramedical staff working in emergency ward of public sector hospitals in Pakistan. Paramedical staff, along with doctors, are the primary human resources who spend the majority of their time serving patients in hospitals to ensure patient health. The emergency department of each hospitals runs two working shifts and previous studies reported that the staff working in emergency ward have faced job burnout, stress, and performance issues due to fatigue and long-hours parallel duties. Based on individual’s working situation, most of the employees are working in the ward to support the families as unemployment is serious issue in Pakistan. In such context, each employee has different motivation level in performing his/her job duties.

We conducted an empirical research using multi-source and multi-wave study in a multi-organization sample of paramedical staff. At the first stage, the authors had a brief dialogue with staff; during this phase, a cover letter was provided to them, which defined the motive for this study, ensured them that their identity would remain confidential, and encouraged their voluntary participation. During this phase, a list of paramedical teams was developed and assigned a unique number to collect and match the respondent and supervisor responses. In next phase, the highly-structured questionnaires were distributed to 250 paramedical staff and 52 supervisors using random sampling technique during January 2020 to April 2020. After multiple follow-ups, a total of 186 questionnaires from the respondents and 44 from supervisors were received. However, due to high missing values, and unmatched questionnaires, 175 subordinates and 40 supervisor responses were matched and processed for further analyses. Overall, there was a 58% response rate in the target sector of this study. The issue of common bias methods was avoided through multi-wave data collections from subordinates and immediate supervisors. At time 1, the subordinates were asked to rate about family motivation, mediating variable – psychological meaningfulness, and control variables. After 4 weeks, the subordinates were asked to rate moderating variable (collectivistic culture) and turnover intentions. After another 4 weeks, the questionnaires were sent to the supervisors to rate the job performance of his/her respective subordinates. Of the responses, 89% were female, and 11% were male. The average age was 24 years, the average working experience was 4 years, and the mean qualification was undergraduate. Lastly, the average group size was 4.375.

### Measures

All the scales have adopted from previous literature with strong reliabilities and validities. The responses were collected on five-point Likert Scale ranging from strongly disagree = 1 to strongly agree = 5.

#### Family motivation

To measure family motivation, this study adopted Menges et al.’s (2017) five-item scale. Sample item of the construct is “*I care about supporting my family*.”

#### Intent to Leave

To measure intent to leave, this study adopted a three-item scale (Vigoda, 2000). Sample item is “My subordinate often thinks about quitting this job.”

#### Job performance

Job performance was measured with a seven-item scale by Williams and Anderson (1991). Sample item of this scale is “My subordinate never fails to perform essentials duties in his/her job.”

#### Psychological meaningfulness

Psychological meaningfulness was measured with a six-item scale (α = 0.92), taken from May et al. (2004). The sample item is “the work I do on this job is worthwhile; my job activities are personally meaningful to me.”

#### Collectivist culture

Collectivism was measured using the six-item scale from previous research of Yoo et al. (2011). Sample item of this instrument is “Individuals should sacrifice self-interest for the family.”

#### Control variables

This study also determines the effect of demographic profile, using one-way ANOVA, as control variables on dependent variables. For this study, the designation, qualification level, institute name, income, shift, and status and gender have significant relation with job performance. For psychological meaningfulness we have found shift, institute name, and no of family member significant. For intent to leave we have found significant impact of shift, institute name, and total number of family members.

## Results

The Table 1 contains the summary of descriptive analysis along with reliabilities, and correlations of all study variables. The table indicates that family motivation positively correlates with psychological meaningfulness (*r* = 0.20**, *p* < 0.01), job performance (*r* = 0.22**, *p* < 0.01) and negatively associates with intention to leave (*r* = −0.20* *p* < 0.01). Similarly, psychological meaningfulness positively correlates with job performance (*r* = 0.28**, *p* < 0.01) and negatively associates with intention to leave (*r* = −0.23**, *p* < 0.01). Additionally, a positively correlation is also exist between collectivistic culture and psychological meaningfulness (*r* = 0.55**, *p* < 0.01).

**Table 1:** Correlations and descriptive statistics.

	Mean	S.D	1	2	3	4	5	6
1. Family motivation	3.85	0.79		**(0.71)**				
2. Job performance	3.97	0.50	0.22^**^		**(0.77)**			
3. Intent to leave	2.39	0.78	−0.20^**^	−0.14		**(0.76)**		
4. Psychological meaningfulness	2.78	1.11	0.20^**^	0.28^**^	−0.23^**^		**(0.90)**	
5. Collectivist culture	3.23	0.96	0.30^**^	0.49^**^	−0.26^**^	0.55^**^		**(0.80)**

*n* = 175.

* *p* < 0.1.

** *p* < 0.01.

Bold values represents AVEs.

This study analyzed the main effects of proposed hypotheses H1, H2, and H3 through multiple linear regression analysis, as presented in Table 2. The first hypothesis of this study stated that employees who are motivated due to family motivation are inclined more towards positive job performance and negatively inclined to leave. The results are in support of H1; there was a positive relationship between family motivation and job performance (B = 0.14, 95% CI [0.236, 0.049] *p* < 0.01) and a negative relationship with the intent to leave (B = −0.20, 95% CI [−0.051, −0.340], *p* < 0.01). As employees are more motivated to support their family, they are more cognitively involved in their jobs, show good performance at work, and have less intent to leave. The results are also in support of the second argument (H2); those employees who are motivated due to family motivation find their jobs more meaningful. Family motivation was found to be positively related to psychological meaningfulness (B = 0.29, 95% CI [0.494, 0.079], *p* < 0.01). Additionally, this study found results in support of the third proposed hypothesis (H3), indicating that employees who perceive their job as more psychologically meaningful show positive job performance (B = 0.13, 95% CI [0.019, 0.062], *p* < 0.01) and probably less intent to leave (B = −0.16, 95% CI [−0.055,-0.258], *p* < 0.01), as represented in Table 2.

**Table 2:** Main effects.

	Psychological meaningfulness			Job performance			Intent to leave		
Predictors	*β*	*R* ^2^	∆*R*^2^	*β*	*R* ^2^	∆*R*^2^	*β*	*R* ^2^	∆*R*^2^
Control variables for psychological meaningfulness^a^, job performance^b^, and intent to leave^c^		0.13			0.13			0.26	
Family motivation	0.29	0.15	0.02	0.14	0.14	0.02	−0.20	0.28	0.02
Control variables for job performance and intent to leave					0.13			0.26	
Psychological meaningfulness				0.13	0.19	0.07	−0.16	0.29	0.03

Control variables for “a” shift, institute and number of family members. Control variables for “b” designation, qualification, institute, income, shift, status and gender. Control variables for “c” shift, institute and total number of family members.

For confirming mediational effects in the proposed research framework, this study used the Preacher and Hayes (2004) bootstrapping method through PROCESS, which helped eliminate the “nuisance in EXP overflow error.” This test helped cater to the indirect effects of family motivation on job performance and intent to leave, countering the effects of statistical problems caused by non-normal or asymmetric sample distribution (Hayes, 2009), with 5,000 random bootstrap samples (Preacher and Hayes, 2004). Table 3 represents the mediation effects of proposed research hypotheses H4 and H5. This study found the presence of mediation effects of psychological meaningfulness on the relationship between family motivation and job outcomes (job performance, intent to leave). Results successfully support H4; the confidence intervals for the indirect effects of family motivation on job performance *via* the psychological meaningfulness results showed the existence of partial mediation (Boot effect = 0.0319, 95% CI [0.0720, 0.0633], *p* < 0.01). For the confirmation of H5, the results showed that the confidence interval for the indirect effect of family motivation on intent to leave under psychological meaningfulness contains zero value, which manifests the insignificant relationship, confirming the existence of full mediation (Boot effect = −0.0385, 95% CI [−0.0905, −0.0085], *p* < 0.01).

**Table 3:** Mediating effect of psychological meaningfulness between family motivation and Job outcomes (job performance and intent to leave).

	B	SE	*t*	*p*	*R* ^2^
**Total effects**					
Family motivation → Job performance	0.14	0.05	3.02	0.00	0.05
Family motivation → Intent to leave	−0.20	0.07	−2.68	0.01	0.04
**Direct effects**					
Family motivation → Job performance	0.11	0.05	2.36	0.02	0.05
Family motivation → Intent to leave	−0.16	0.07	−2.14	0.03	0.04
**Indirect effects**		**Boot effect**	**Boot SE**	**Boot LLCI**	**Boot ULCI**
Family motivation → Psychological meaningfulness → Job performance		0.0319	0.02	0.0633	0.0720
Family motivation → Psychological meaningfulness → Intent to leave		0.0385	0.02	0.0905	0.0085

B= Unstandardized coefficients. No. of bootstrap sample = 5,000. CI = 95% of confidence interval.

**Figure 1:**
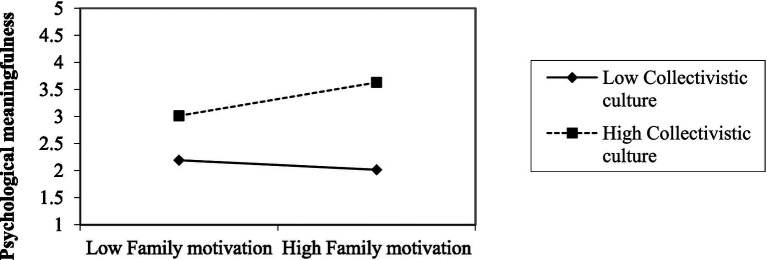
Moderating effect of collectivist culture on the relationship between family motivation and psychological meaningfulness.

**Figure 2:**
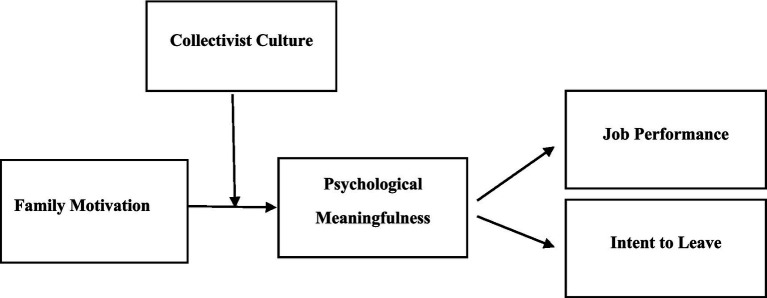
Conceptual model.

For confirming the moderating effect of a collectivist culture on the relationship between family motivation and psychological meaningfulness, this study tested the effects of the interactional term of family motivation × cultural collectivism to predict employees’ psychological meaningfulness in their jobs. The moderation was checked by PROCESS method as proposed by Preacher and Hayes (2004). This study found significant positive results for the proposed interactional effects (β = 0.26, *p*<0.01). For further clarification, the graphical representation (Figure 1) confirms the interactional effects of collectivistic culture, along with family motivation, for the relationship between family motivation and psychological meaningfulness. The graph depicts the level of an employee’s perception of psychological meaningfulness for family motivation on high and low levels of collectivism in Figure 1, combined with a simple slope analysis. As shown in Figure 2, the graphic representation highlights the impact of a collectivistic culture on the relationship between family motivation and employee psychological meaningfulness; these results depict the support of proposed hypothesis H6. The conceptual framework of this study is presented in Figure 1. Individuals in a highly collectivistic culture identify themselves more by their family relationships. Profoundly, their activities and actions are for the betterment of their family structure (Islam, 2004), so they find their work more meaningful because, ultimately, they have to support and gratify their family responsibilities. Thus, people capture more psychological meaningfulness in their jobs when in a collectivistic culture; family motivation acts more saliently, which boosts the meaning of assigned workplace duties (Table 4).

**Table 4:** Moderating effect of collectivist culture between family motivation and psychological meaningfulness.

	B	SE	*t*	*p*	*R* ^2^	LLCI	ULCI
Family motivation	−0.7	0.29	−2.43	0.02	0.33	−1.26	−0.13
Collectivist culture	−0.37	0.36	−1.01	0.31		−1.09	0.35
Interactional effect of collectivist culture and FM	0.26	0.09	2.78	0.01		0.08	0.44

## Discussion

This research work anticipated the impact of family motivation as a source of psychological meaningfulness for individuals living in the collectivistic society of Pakistan. As evidenced by the literature, limited research has been done on family motivation (Menges et al., 2017; Tariq and Ding, 2018), and previous research (Rosso et al., 2010; Liu et al., 2020; Zhang et al., 2020) manifested only a few studies exploring the role of the family as a source of meaning in one’s work.

This study found significant positive relationships (H1, H2, and H3) between family motivation and job performance and psychological meaningfulness and a negative relationship with the intent to leave, which indicates that individuals feel more responsibilities to serve their families. Therefore, individuals may feel more meaning in their work, generating good job performance and less intent to leave an organization. Wang and Lin (2018) find positive association of different motivation factors (such as job autonomy, and skill variety) increases the meaningfulness. Fowler (2014) claims that individuals align their values and objectives of serving their families with work to find meaning. In this way, they find the reason “why” and bear any hardships in the form of “how” in the course of accomplishing those aims of life (Frankl, 1984). Meaning can be constructed individually by building perceptions through social norms and shared perceptions, or it can be mutually influenced by both of them (Pratt and Ashforth, 2003). In the literature, experiencing meaningfulness in work mostly depicted a positive valence; under this scene, an individual’s work experience holds a greater amount of significance and positive meaningfulness (Hackman, 1980).

The cognitive view of motivation has been followed by SDT (Ryan and Deci, 2000), which explains the purest form of motivation generated when an individual experiences three psychological conditions (autonomy, relatedness, and competence) in their work activities. This view established a prominent foundation in organizational behaviors (Seo et al., 2004) and literature on the meaning of work. This study rationalized that individuals have a free choice to make decisions and set goals (to serve family) because they feel more relatedness towards their loved ones (family members). They intuitively capture the meaning of work in the form of accomplishing family responsibilities. Thus, they do a better job in the workplace and are less likely inclined to leave a firm. Family motivation is a strong motivational factor (Menges et al., 2017; Tariq and Ding, 2018) for individuals, so they carry their professions with commitment.

Further, this study found significant relationships (H4, H5 and H6) for the proposed hypotheses, indicating that psychological meaningfulness represents a key mechanism by which family motivation acts more saliently, translating into high job performance and less intent to leave an organization. Motivational triggers are crucial for organizations to generate higher employee performance (Ghaffari et al., 2017); without motivation, employees do not give their best. This situation is more difficult in developing countries (Seniwoliba and Nchorbono, 2013). Thus, family motivation (Menges et al., 2017; Tariq and Ding, 2018; Liu et al., 2020; Zhang et al., 2020 can trigger more meaning for one’s job (Rosso et al., 2010), which directly impacts job performance and inclination to leave an organization.

Moreover, this study incorporated the interactive impact of a collectivistic culture on the relationship between family motivation and psychological meaningfulness. Baumeister (2018) highlights that social relationships and meaningfulness are significantly associated with each other, and that meaning is somewhat more salient in specific cultural contexts. Authors suggest that work is deemed more meaningful when social or cultural boundaries ascribe values to the work activities (Martela and Riekki, 2018). The family and work are two parallel factors for an adult that has significant role in shaping family motivation. Pakistan, being a collectivistic society, the employees are adhering family ties despite of work-related issues. Rastogi et al. (2019) argue that favorable perceptions regarding family and work has been generated through motivational factors such as pay.

In collectivistic cultures, values and norms align individuals’ behaviors with the compulsion to devote oneself to a social circle and provide benefits to one’s family (To et al., 2014). Thus, collectivistic culture acts as a source of meaningfulness (Islam, 2004) alongside family motivation (Menges et al., 2017) and generates positive interactive consequences for the relationship between family motivation and psychological meaningfulness. Individuals in the collectivistic culture of Pakistan give more importance to their relationships with family and kinship structure, and their actions are based on the well-being of these structures (Islam, 2004). So, in the presence of a collectivist culture, family motivation acts more saliently and boosts the meaningfulness of an employee’s work. To cater to family responsibilities, individuals sacrifice themselves and prioritize the wellness of family members (Yoo et al., 2011). Consistent with SDT (Ryan and Deci, 2000), individuals give more importance to their family relationships; in a collectivistic culture, employees think family is their responsibility. By serving this responsibility, individuals assume that they are contributing towards family; this contributing sense provides more perceived meaningfulness in a job. McManus (2017) claims that “connecting the dots” support to align family motivation with work meaningfulness (Sarwar et al., 2020); the author said that working parents, regardless of their work nature, find the meaning in their work only because they have to support their family. Hence, they feel more attachment to their work, which ultimately leads to positive job outcomes.

### Theoretical implications

Brief and Nord (1990) in their seminal research book “On Meaning of Work,” spotlight how family influences one’s meaning of work. First, putting a strain on work through demands in the form of economic resources, time, and energy provides the conception of individuals fulfilling the demands of their family through economic rewards. In this case, the economic rewards act more saliently and probably associate economic meaning with work activities (Brief et al., 1997). In contrast, a family may serve as a source of motivation, providing a supportive, relaxing environment for recovery from the demands of a job by acknowledging the role of work in one’s life and expressing admiration for respect, love, money, labor, assistance, information, etc. Thus, both spheres shape each other for the conception of meaning in work activities. In a broader sense, many individuals carry their jobs intending to improve the quality of life of their families (Bullock and Waugh, 2005).

In this study, family motivation and collectivistic culture served as a window for ascribing the meaning of work. Social, cultural, and individual values, beliefs, and motivations have a strong impact on how individuals ascribe meaning and associate it with the significance of work activities (Greenhaus and Powell, 2006). Kwan et al. (2021) contend that the sense of belongingness (especially in collectivistic societies) maintain healthy relationships, reduces the subordinate-manager conflict, and increases the in-role and extra-role performance. Regarding interaction, relationships with others (family) and groups within or outside the work setting influence the meaning of work activities (Pratt and Ashforth, 2003). Overall, this research work provided a foundation for comprehending the consequences of family motivation on an individual’s work meaningfulness and job outcomes. This study adds to the literature by specifying the role of a family as a source of motivation and psychological meaningfulness in an employee’s work life, consequently leading to higher job performance and decreased intent to leave and by revealing how family motivation acts more saliently in the presence of a collectivist context for pulling a sense of meaningfulness into one’s work.

### Practical implications

This study provides several key insights for organizations that hope to maintain high job performance and low intent to leave among their employees. A handful of research studies explore the crucial role of motivational triggers on enhancing and maintaining employee job performance (Ghaffari et al., 2017), countering turnovers, as this phenomenon is more severe in underdeveloped countries. The dilemma of low effectiveness and productivity dominantly prevails in impoverished areas because employees work in rough and tough conditions (Davis, 2010). Past research studies prove that rotating schedules among the paramedical staff working in the USA, Taiwan, and Iran negatively affect their lives, so they are unable to manage their work and family responsibilities (Battu and Chakravarthy, 2014; Raza et al., 2022). Paramedical staff work in a challenging, critical environment and work overnight, so they experience sleep deprivation, fatigue, and stress, resulting in impatience, medical errors in health care, and lower performance (Battu and Chakravarthy, 2014). A supervisor should highlight these issues in front of higher authorities, so they comprehend the actual situations and take the initiative to resolve these problems as soon as possible.

Many other pedagogical implications emerged from the findings of this study, which are also relevant to cultural contexts other than Pakistan. Thus, a family has a strong influence on motivation and serves as a source of motivation, which pulls individuals towards their job, regardless of stressful work activities, by conveying meaning to that job. In doing so, they cognitively capture the sense of meaning and identify themselves towards their families. Moreover, being in a collectivistic culture, they feel more responsible for contributing to them and, consequently, are more engaged with their jobs. This study highlights the role of the family as a powerful source of generating meaning for employees in their work, so employers should acknowledge the importance of an employee’s family and align their organizational policies with them to spur their best potential at work. Over time, the world is becoming a more global village; work and family life are blurring together, disentangling the impact of time and space, so individuals can interact and communicate frequently and perform their duties at any place. In this competitive environment, most firms now recognize the need to retain and maintain their talented pool of employees who offer their best qualities to cater to survival and growth opportunities for the organization (Ruslan et al., 2014).

### Limitations and future research directions

This research study has some limitations that offer opportunities for further research studies. This study is cross-sectional, as the authors proposed that meaningfulness and motivation suffer a cross-sectional barrier. So, investigations in longitudinal or experimental research designs are encouraged because, with time, families extend or children grow up, and their tuition fees increase, so the results may differ comparatively with current results. Second, this study used a small sample size and restricted to paramedical staff only. So instead of specifically paramedical staff, in the future, this phenomenon should be studied in retail, banking, and textile sectors with a large sample size. Third, this study only highlighted the interactional effects of a single collectivist culture (Hofstede, 2011) for comprehending the phenomenon of meaningfulness in one’s work. There could be other social and cultural forces and environments that strongly influence an individual’s perception; individuals must decide for themselves what is or is not meaningful. The seminal work of the researchers enforced the importance of cultural and social factors (Geertz, 1973) that impact the perception of meaningfulness and expand the knowledge to comprehend how others, cultural, or personal norms matter more for meaning conception (Geldenhuys et al., 2014).

Most of the time, family work is depicted in the negative spectrum (Liu et al., 2020; Zhang et al., 2020); limited research explores its positive side (Menges et al., 2017; Tariq and Ding, 2018; Erum et al., 2020), so there is room for cultivation in this domain. There is a need to investigate its antecedents and consequences. Individual differences should be included as a moderator, for example, self-efficacy; when individuals feel that they are making a change or contributing something towards coworkers, family, a group, or the organization, they feel they are affecting positive impacts and experience more meaningfulness in their work. The work-family psychological contract can be seen as a mediator between family motivation and employee job outcomes.

## Data availability statement

The raw data supporting the conclusions of this article will be made available by the authors, without undue reservation.

## Ethics statement

Ethical review and approval was not required for the study on human participants in accordance with the local legislation and institutional requirements. The patients/participants provided their written informed consent to participate in this study.

## Author contributions

All authors listed have made a substantial, direct, and intellectual contribution to the work and approved it for publication.

## Conflict of interest

The authors declare that the research was conducted in the absence of any commercial or financial relationships that could be construed as a potential conflict of interest.

## Publisher’s note

All claims expressed in this article are solely those of the authors and do not necessarily represent those of their affiliated organizations, or those of the publisher, the editors and the reviewers. Any product that may be evaluated in this article, or claim that may be made by its manufacturer, is not guaranteed or endorsed by the publisher.
